# Impact of specific productivity and operation mode upon the biophysical properties of HIV-1 Gag-based virus-like particles

**DOI:** 10.1007/s00253-025-13560-9

**Published:** 2025-08-08

**Authors:** Pol Pérez-Rubio, Elianet Lorenzo Romero, Josefina Casas, Andy Díaz-Maneh, Francesc Gòdia, Laura Cervera, Jesús Lavado-García

**Affiliations:** 1 Grup d’Enginyeria de Bioprocessos I Biocatàlisi Aplicada, Universitat Autònoma de Barcelona, Escola d’EnginyeriaCampus de Bellaterra, Cerdanyola del Vallès, 08193 Barcelona, Spain; 2 Research Unit On Bioactive Molecules (RUBAM), Department of Biological Chemistry, Institute for Advanced Chemistry of Catalonia (IQAC-CSIC), Spanish National Research Council (CSIC), 08304 Barcelona, Spain; 3 Centro de Investigación Biomédica en Red de Enfermedades Hepáticas y Digestivas (CIBEREHD), Instituto de Salud Carlos III, 28029 Madrid, Spain; 4 Group of Mammalian Cell and Bioprocess Engineering, DTU Biosustain, Novo Nordisk Foundation Center for Biosustainability, Technical University of Denmark, 2800 Kgs. Lyngby, Denmark

**Keywords:** Virus-like particles (VLPs), TGE, HEK293, Lipidomics, Perfusion, Bioprocess development, Functionalization, ATM

## Abstract

**Abstract:**

Virus-like particles (VLPs) are non-infective vaccine candidates that have gained interest given their natural ability to elicit strong immune responses. Particularly, HIV-1 Gag-based VLPs are one of the most described platforms for vaccine development, provided their ability for successful pseudotyping either by genetic engineering or click chemistry. When Gag polyprotein is recombinantly expressed, VLPs are naturally assembled in the vicinity of the cell membrane and then secreted by cell budding, taking part of the host cell membrane. Their properties are dependent upon the cell line and manufacturing method. Although great advancements toward the implementation of analytical methods have been made, VLP quality attributes are quite unclear whenever production is enhanced by metabolic engineering or process intensification strategies. This work offers a comparative study of VLP quality attributes upon transient gene expression (TGE) in HEK293 cell cultures operated in batch and perfusion mode. Moreover, the impact of specific productivity is also studied by ataxia telangiectasia mutated (ATM) gene silencing, which has been reported to enhance fourfold VLP production. A linear negative correlation was found between the ratio of Gag monomers/VLP and specific productivity. 3100 ± 100 monomers/VLP were obtained for the standard batch production, dropping to 1900 ± 100 and 800 ± 60 for the perfusion and batch ATM-knockdown conditions, respectively. Furthermore, functionalization rates were measured in terms of Cy5 per total particles (TP). Both perfusion-derived nanoparticles achieved functionalization rates of 2800 Cy5/TP. On the contrary, those nanoparticles produced in batch yielded functionalization rates below 1000 Cy5/TP. Moreover, a complete lipidome analysis revealed a relative decrease in the quantity of lipid/particle for all studied conditions in comparison to the standard batch production. Finally, all VLP samples were characterized to assess the impact of the differential physicochemical properties upon purification and stability rates.

**Key points:**

• *VLP quality inversely correlates with Gag-specific productivity and operation mode.*

• *Functionalization and lipid content drop with metabolic burden or ATM silencing.*

• *Perfusion enables high VLP recovery and lyophilization with preserved morphology.*

**Supplementary Information:**

The online version contains supplementary material available at 10.1007/s00253-025-13560-9.

## Introduction

Vaccines based on virus-like particles (VLPs) have gained interest over the past few years due to their intrinsic ability to induce strong humoral and cellular responses (Fuenmayor et al. 2017; Nooraei et al. 2021). They consist of self-assembled multimeric nanostructures that highly resemble the native structure of the virus but lack the genetic material to trigger infection (Mohsen and Bachmann 2022; Tariq et al. 2022). In the case of human immunodeficiency virus type 1 (HIV-1) Gag VLPs, these particles are composed of monomers of the structural Gag polyprotein, bounded by cell membrane, as they are secreted by cell budding (Cervera et al. 2019; Lavado-García et al. 2021b; Martins et al. 2022). When recombinantly expressed, Gag monomers are transported to the cell membrane where the endosomal sorting complex required for transport (ESCRT) coordinates their assembly, oligomerization, and finally facilitates their excision from the plasma membrane (Jun et al. 2008; Lavado-García et al. 2021a). Such characteristics can be further exploited for particle pseudotyping either by genetic means or by click chemistry (Kueng et al. 2011; Patel and Swartz 2011; Ho et al. 2012; Lasickiene et al. 2012; Brune and Howarth 2018; Soares et al. 2019). For instance, this has been proven successful to produce SARS-CoV-2 and avian influenza Gag VLPs (Haynes et al. 2009; Boix-Besora et al. 2022). Mammalian cells are the preferred platform for the manufacturing of such VLPs provided they possess the required cellular machinery for proper particle budding and can perform complex post-translational modifications (Zhu 2012). One approach to producing Gag VLPs in mammalian cells is by transient gene expression (TGE) which involves the deliberate introduction into cell cultures of the plasmid coding for the gene to be expressed (Cervera et al. 2013). When produced in batch, TGE allows production of reasonable amounts of recombinant protein in a relatively short time span that avoids the high time-consuming task of developing stable cell lines (Baldi et al. 2007; Nettleship et al. 2015; Gutiérrez-Granados et al. 2018). Besides, recent advances in bioprocess engineering have permitted the implementation of an alternative perfusion system using a protocol of extended gene expression (EGE) based on subsequent retransfections and continuous media replacement. This extends production time, thereby increasing volumetric productivity (Cervera et al. 2015; Lavado-García et al. 2020a). Moreover, specific productivity in both batch and perfusion can be enhanced by gene silencing strategies involving the use of chemical additives or short hairpin RNAs (shRNAs) (Fuenmayor et al. 2018). The high flexibility that TGE offers currently makes it an attractive strategy for the manufacturing of recombinant proteins in several cell lines (Derouazi et al. 2004; Wright 2009; Gutiérrez-Granados et al. 2016; Jain et al. 2017). Most of the reported studies propose mechanisms to increase VLP production levels, yet few are devoted to understanding what these intensifications convey in terms of product quality. In these lines, it has been studied that substantially increasing productivity negatively influences critical quality attributes (CQA) of many biopharmaceutical processes (Brunner et al. 2017; Mulukutla et al. 2017; Kuang et al. 2021). For instance, defects in antibody glycosylation and native conformation tend to appear when specific productivity surpasses certain thresholds (Torkashvand et al. 2015; Niu et al. 2018; Sissolak et al. 2019). Regarding Gag VLPs, recent quality study assesses Gag stoichiometry indicating that approximately from 3000 to 4000 monomers are assembled into VLPs when produced in HEK293 by TGE while this number is reduced to around 2000 when produced in a HEK293 stable producer cell line, highlighting the importance of the production platform (Lavado-García et al. 2021b). Furthermore, other studies point out the ultrastructural differences between Gag VLPs produced in HEK293 or insect cells by TGE (González-Domínguez et al. 2020). Although progress has been made, VLP quality attributes remain largely unexplored. To the best of our knowledge, there are no reported studies that examine how specific productivity and the subsequent reduction in the number of monomers may impact the quality and stability of Gag-based VLPs. Hence, this work proposes easy-to-implement analytical tools to measure VLP quality attributes that are potentially critical in process development. The impact of specific productivity upon VLP production by TGE in HEK293 cells is studied by ataxia telangiectasia mutated (ATM) silencing, which has been reported to increase titers by fourfold (Díaz-Maneh et al. 2025). Likewise, the impact of perfusion implementation using EGE is also explored, allowing a double comparison. Resistance to ultracentrifugation, monomer/VLP determination, lipidic characterization, rate of functionalization, and freeze-drying stability are all studied to establish possible differences that may arise from enhanced levels of productivity and from diverse modes of operation. All in all, the work presented broadens the knowledge in VLP characterization and settles techniques for routine quality assessment of VLP and co-produced extracellular vesicles (EVs).

## Materials and methods

### Cell line and culture conditions

The experimental study employed a serum-free, suspension-adapted HEK293SF-3F6 cell line, derived from human embryonic kidney 293 (HEK293) cells and obtained from the National Research Council of Canada (NRC, Montreal, Canada), kindly provided by Dr. Amine Kamen. The cells were routinely cultured in disposable polycarbonate vented shake flasks (Corning, NY, USA) with volumes of 125 mL, 250 mL, and 1000 mL. The flasks were placed on a Kuhner shaker LT-X (Kuhner, Birsfelden, Switzerland) and incubated at 37 °C, 5% CO_2_, and 85% relative humidity (RH) and a shaking speed of 130 rpm. To maintain the cells in exponential phase, regular passages were performed every 2–3 days, maintaining cell densities ranging from 0.3 to 0.5 × 10^6^ cells/mL with viabilities above 95%. The culture medium used was HyCell TransFx-H from HyClone (GE Healthcare, Chicago, IL, USA), supplemented with 4 mM GlutaMAX (Gibco, Carlsbad, CA, USA) and 0.1% Pluronic F-68 (Gibco).

Cell concentration and viability were determined using a NucleoCounter NC-3000 automatic cell nuclei counter (Chemometec, Allerod, Denmark), following the manufacturer’s instructions.

### Transient transfection and protein expression

Standard transfections were performed at a cell density of 2 × 10^6^ cells/mL and a DNA concentration of 1 µg/mL. The cationic transfection reagent utilized in this study was PEIPro (PolyPlus, Illkirch-Graffenstaden, France). In brief, the appropriate amount of DNA was added to fresh culture medium (10% of the total culture volume to be transfected) and vortexed for 10 s. Then, polyethyleneimine (PEI) was added in a ratio of 2:1 (w/w) with respect to DNA and subjected to vortexing 3 times for 3 s, followed by a 15-min incubation at RT. Finally, the transfection mixture was added to the respective cultures.

Two different plasmids were employed in the experiments: pGag::eGFP (Venereo-Sanchez et al. 2016) and pGag::eGFP-shATM (Díaz-Maneh et al. 2025). Both encoded the HIV-Gag polyprotein fused in-frame with the enhanced green fluorescent protein (eGFP) and contained the same CMV enhancer and CMV promoter. pGag::eGFP-shATM also encoded a short-hairpin RNA (shRNA) sequence under the pU6 promoter targeting the ATM gene. Regarding batch experiments, cell cultures were harvested at 72 h post transfection and centrifuged at 3000 g for 10 min. Supernatants were stored at − 80 °C until use. Perfusion supernatants were generated as previously described (Lavado-García et al. 2020a). Briefly, perfusion was achieved using an alternating tangential flow (ATF) cell retention device (Repligen, Waltham, MA, USA) with 0.2 µm pore size and 0.13 m^2^ of filtration area hollow fiber modules (Repligen, Waltham, MA, USA) and an ATF flow rate of 0.6 L/min. When performing transfection, perfusion was stopped to incubate the cells with the DNA/PEI solution and reestablished 2 h after transfection. Twenty-four hours after the first transfection, a second transfection was carried out, following the optimized EGE protocol. To carry out media replacement, the filtration rate was set at 0.26 mL/min at the beginning of the process and modified every day depending on the viable cell density to maintain a cell-specific perfusion rate (CSPR) of 30 pL/cell/day.

### HIV-1 Gag VLP quantification – fluorimetry

To determine the concentration of HIV-1 Gag::eGFP virus-like particles (VLPs), a validated quantification assay based on fluorimetry was employed (Lavado-García et al. 2021b). The VLP-containing supernatants were obtained by centrifuging at 1000 g for 5 min. GFP intensity, an indicator of VLP concentration, was measured using a Cary Eclipse fluorescence spectrophotometer (Agilent Technologies, Santa Clara, CA, USA). Relative fluorescence units (RFUs) of transfected samples were calculated by subtracting the readings from the non-transfected control. To convert RFU values into VLP concentration, the following correlation was applied: 

1 $$VLPs/mL=\:(4.448\times RFU-63.3)\times10^8$$

### HIV-1 Gag VLP quantification – flow virometry (FV)

HIV-1 Gag::eGFP VLPs were quantified utilizing a CytoFLEX LX flow cytometer (Beckman Coulter, Brea, CA, USA). VLPs were detected based on violet side scatter (V-SSC) and FITC fluorescence signals. Laser gains were set to 72 for forward scatter (FSC), 135 for side scatter (SSC), 9 for V-SSC, and 500 for FITC. Prior to analysis, samples were appropriately diluted in filtered PBS to achieve a concentration range of 500 to 5000 events/µL, ensuring an abort rate below 5%. A minimum of 20,000 VLP events were recorded at a flow rate of 10 µL/min. VLPs were distinguished from background noise using V-SSC vs B525-FITC density plots. Results were normalized employing an internal control. Data analysis was conducted using CytExpert v.2.3 software (Beckman Coulter, Brea, CA, USA).

### HIV-1 Gag VLP quantification by ELISA

The concentrations of Gag::eGFP polyprotein were determined using a p24 enzyme-linked immunosorbent assay (ELISA) employing the commercially available Innotest® HIV antigen mAb kit (Innogenetics NV, Ghent, Belgium). The assay procedure followed the instructions provided by the manufacturer to ensure accurate results.

### HIV-1 Gag VLP and extracellular vesicle quantification – nanoparticle tracking analysis (NTA)

Quantification of both fluorescent and non-fluorescent diffracting particles was performed using Nanoparticle Tracking Analysis (NTA). The measurements were conducted using a NanoSight LM 20 Device (NanoSight Ltd., Amesbury, UK) equipped with a blue laser (488 nm) for quantifying Gag::eGFP VLPs and a neutral density filter for assessing total particle concentration through light scattering. Each value represents the average of three independent measurements. Data analysis was carried out using NanoSight NTA 3.1 software (Malvern Panalytical Ltd., Malvern, UK).

### Ultracentrifugation

Concentrated and purified HIV-1 Gag virus-like particles (VLPs) were obtained through a single-cushion ultracentrifugation method. To summarize, a clarified supernatant from a HEK293 cell culture that underwent transient transfection was layered onto a 30% sucrose cushion (3 mL) and centrifuged at 31,000 rpm for 2 h at 4 °C using a SW32 rotor in a Beckman Optima L100XP centrifuge (Beckman Coulter, Brea, CA, USA). Following ultracentrifugation, the supernatant was discarded, and the resulting pellet was resuspended in 1 mL of PBS (GE HealthCare, Chicago, IL, USA). The concentrated material was then stored at − 80 °C for further experimentation.

### Click chemistry functionalization of Gag VLPs

Copper-free click chemistry method was utilized to functionalize membrane proteins of purified VLPs coming from ultracentrifugation with Cy5 in a two-step reaction. For primary activation, 5 × 10^11^ total particles of each sample were incubated for 24 h at 37 °C under constant mixing with 55 µM dibenzocyclooctyne-sulfo-*N*-hydroxysuccinimidyl ester (DBCO, Sigma Aldrich, St. Louis, MO, USA). Then, 30 µM Cy5-azide (Sigma Aldrich, St. Louis, MO, USA) was added to the mixture and maintained under the same conditions for 12 h. Unconjugated reagent was removed by performing a secondary ultracentrifugation in the conditions abovementioned. Cy5 molecules/VLP were assessed by in-house validated fluorometric assay (García-Trujillo et al. 2025).

### Clarification, purification, lyophilization, and HPLC analysis

In short, all cell culture supernatants were clarified with Supracap™ 50V100™ depth filter capsules (Pall Corporation, Port Washington, NY, USA). Each filter was pre-equilibrated with PBS (Hyclone) before filtration. Then, the clarified supernatants were loaded into a 4.7 mL prepacked HiScreen™ CaptoQ™ ImpRes (GE Healthcare, Chicago, IL, USA) automatically operated by an AKTA Pure system (GE Healthcare, Chicago, IL, USA). Both purification protocols have been previously described (Lorenzo et al. 2023). VLP lyophilization was performed according to a previously reported study (González-Domínguez et al. 2021). High-performance liquid chromatography (HPLC) analyses were conducted with an Agilent 1100 Series HPLC System (Agilent Technologies, Santa Clara, CA, USA) equipped with an Agilent AdvanceBio SEC 2.7 µm (Agilent Technologies, Santa Clara, CA, USA) at Servei d’Anàlisi Química (SAQ) of UAB (Barcelona, Spain). Clarified samples were directly plunged provided column equilibration. Recorded signal from PBS was taken as blank.

### dsDNA and total protein quantification

The concentration of host cell DNA was determined using the Quant-itTM Picogreen dsDNA assay kit (Sigma Aldrich, San Luis, MO, USA), following the manufacturer’s instructions. In summary, serial dilutions of both the standard and samples were prepared in 1X TE buffer and dispensed into 96-well microplates. Subsequently, 100 µL of the diluted Quant-iTTM PicoGreen® reagent (1:1000 dilution) was added to each well, followed by incubation for 5 min at room temperature. The dsDNA standard curve covered a range of 1.5 to 500 µg/mL, and absorbance readings were taken using the Victor 3 microplate reader (Perkin Elmer, Waltham, MA, USA) before and after the addition of the Quant-iTTM PicoGreen® reagent, as the HIV-1 Gag::eGFP VLPs emit in a similar range. The excitation wavelength was set to 480 nm, and the emission wavelength was set to 520 nm. The DNA concentration of the samples was determined by referencing the standard curve and subtracting the background fluorescence. The concentration of host cell protein was determined using the Micro BCA protein assay kit (Thermo Fisher Scientific, Waltham, MA, USA), following the manufacturer’s instructions. To summarize, serial dilutions of both the standard and samples were prepared in phosphate-buffered saline (PBS) and dispensed into the wells of a microplate. Next, 150 µL of the Micro BCA working reagent was added to each well, and the plates were incubated for 1 h at 37 °C. The bovine serum standard curve covered a range of 1.5 to 200 µg/mL, and the absorbance was measured at 562 nm using the Victor 3 microplate reader. The protein concentration of the samples was determined by comparing the absorbance readings to the standard curve.

### Dynamic light scattering

The size distribution of nanoparticles was assessed using dynamic light scattering (DLS) with a Zetasizer Nano ZS Instrument (Malvern Instruments, Malvern, UK). The measurements were performed at a temperature of 25 °C and a viscosity of 0.8872 cP. The instrument utilized a He/Ne 633 nm laser and was set at an angle of 173° for data acquisition. Samples were manually introduced into disposable 1 mL cuvettes (Scharlab S.L., Barcelona, Spain) for analysis. Each sample was automatically measured three times to evaluate the measurement error of the instrument.

### VLP visualization in cryo-TEM

The morphology and electron density of HIV-1 Gag VLPs were investigated under cryogenic conditions. The samples were rapidly frozen by plunging them into liquid ethane at a temperature of − 180 °C. Approximately 2 µL of the sample was then applied to holey carbon grids that had been pre-treated with glow discharge using a PELCO easiGlow discharger unit (Ted Pella Inc., Redding, CA, USA). The cryo-frozen samples were transferred to a Leica EM GP cryo workstation (Leica Microsystems AG, Wetzlar, Germany) and subsequently examined using a JEM-2011 electron microscope (JEOL Ltd., Tokyo, Japan) operating at an acceleration voltage of 200 kV. Throughout the imaging process, the temperature was maintained at − 180 °C by the continuous addition of liquid ethane. Micrographs were acquired using a CCD-multiscan camera (Gatan Inc., Pleasanton, CA, USA) for further analysis and characterization.

### Confocal microscopy imaging

Visualization of VLPs was performed using a LEICA TCS SP8 instrument (Leica Microsystems AG, Weztlar, Germany) equipped with a HyVolution module to enable super resolution imaging. Excitation/emission parameters of each dye used for confocal microscopy were 488 nm/510 nm for GFP and 633/650–795 nm for Cy5. Briefly, 10 µL of each corresponding sample was placed in a glass-mounted slide (Invitrogen, Thermo Fisher Scientific, Carlsbad, CA, USA) and observed under the microscope.

### Western blot and SDS-PAGE

For sample preparation, 40 µL of the sample was mixed with 20 µL of 4X LDS (Sigma Aldrich, St. Louis, MO, USA) sample buffer and 7 µL of 2 M dithiothreitol (DTT) (Sigma Aldrich, St. Louis, MO, USA). The mixture was then incubated at 96 °C for 20 min. The prepared samples were stored at 4 °C until the next step. For gel electrophoresis, 20 µL of each sample was loaded onto precast NuPAGE Bis/Tris gels (Invitrogen, Carlsbad, CA, USA) with a concentration range of 4–12%. To monitor low molecular weight, 5 µL of SeeBlue® Plus2 Prestained Protein Standard (Invitrogen, Carlsbad, CA, USA) was included. Gels were run at 200 V and 400 mA for 45 min in MES-SDS running buffer. Coomassie Brilliant Blue G-250-based EZBlueTM Gel Staining Reagent (Sigma Aldrich, St. Louis, MO, USA) was used for protein staining in the SDS-PAGE gels. For western blot analysis, the proteins were transferred onto 0.2 µm nitrocellulose membranes using the Trans-Blot® turbo system (Bio-Rad Laboratories, Hercules, CA, USA). The membranes were blocked with 5% (w/v) non-fat dry milk in PBS for 30 min, followed by washing with PBS containing 0.1% (w/v) Tween-20. Next, the membranes were incubated overnight at 4 °C with the primary monoclonal antibody against HIV-1 p24 (1:2000) (Icosagen AS, Tartu, Estonia). After washing, immunodetection was performed using an anti-mouse IgG antibody conjugated with a horseradish peroxidase (dilution 1:5000 in PBS 1X) (Bio-Rad Laboratories, Hercules, CA, USA), incubated for 2 h at room temperature, and washed with PBS 0.1% (w/v) Tween-20. Protein bands were visualized by incubating the membranes with a Clarity™ Western ECL Substrate solutions for 2–3 min, and scans were taken in a ChemiDoc MP (Bio-Rad Laboratories, Hercules, CA, USA).

### Lipidomic analyses

#### Glycerolipids and neutral lipids

For sample preparation, 750 µL of a chloroform–methanol solution (2:1, v/v) were added along with 0.01% butylated hydroxytoluene (BTH) as a preservative. The solution contained internal standards provided by Avanti Polar Lipids (Croda International, Snaith, UK), including 16:0 D31_18:1 phosphocholine, 16:0 D31_18:1 phosphoethanolamine, 16:0 D31_18:1 phosphoserine, 17:0 lyso-phosphocholine, 17:1 lyso-phosphoethanolamine, 17:1 lyso-phosphoserine, 17:0 D5_17:0 diacylglycerol, 17:0/17:0/17:0 triacylglycerol, and C17:0 cholesteryl ester, each at a concentration of 0.2 nmol. The samples were thoroughly mixed by vortexing and sonication until they achieved a dispersed state. Subsequently, the samples were extracted at a temperature of 48 °C and left to cool overnight. After cooling, the samples were evaporated until dryness and stored at a temperature of − 80 °C until further analysis. Prior to the analysis, 150 µL of methanol was added to the dried samples, followed by centrifugation at 13,000 g for 5 min. The resulting supernatant was then carefully transferred into UPLC vials, ready for injection.

### Sphingolipids

To analyze sphingolipid quantities, 750 µL of a methanol-chloroform solution (2:1, v/v) containing specific internal standards, including *N*-dodecanoylsphingosine, *N*-dodecanoylglucosylsphingosine, *N*-dodecanoylsphingosylphosphorylcholine, and C17-sphinganine at a concentration of 0.2 nmol each from Avanti Polar Lipids, was combined with 0.05 mL of serum and the sample of interest. The extraction process was carried out by maintaining the samples at a temperature of 48 °C for an extended period overnight, followed by cooling. Seventy-five microliter of 1 M KOH in methanol was added to the samples, and the entire mixture was incubated at 37 °C for 2 h. After the incubation, 75 µL of 1 M acetic acid was added to the samples to adjust the pH. The samples were then subjected to evaporation until complete dryness, preserving them at − 20 °C until analysis. Prior to it, the dried samples were reconstituted by adding 150 µL of methanol. The reconstituted samples were subjected to centrifugation at a speed of 13,000 g for 5 min to separate the supernatant from any remaining sediment or particles. From the resulting supernatant, 130 µL were carefully transferred to a new vial prepared for injection.

#### LC-HRMS

LC-HRMS analysis was performed using an Acquity ultra high-performance liquid chromatography (UHPLC) system (Waters, Milford, MA, USA) coupled with a time-of-flight detector (LCT Premier XE). Full scan spectra were acquired from 50 to 1800 Da, with data points collected every 0.2 s. Mass accuracy was maintained at a resolving power of 10,000 using an independent reference spray via the LockSpray interference. Lipid extracts were injected onto an Acquity UHPLC BEH C8 column (1.7 µm particle size, 100 mm × 2.1 mm; Waters, Milford, MA, USA) at a flow rate of 0.3 mL/min and a column temperature of 30 °C.

The mobile phases consisted of methanol with 2 mM ammonium formate and 0.2% formic acid (A) and water with 2 mM ammonium formate and 0.2% formic acid (B). A linear gradient elution program was utilized: 0.0 min: 20% B; 3 min: 10% B; 6 min: 10% B; 15 min: 1% B; 18 min: 1% B; 20 min: 20% B; 22 min: 20% B. Positive identification of compounds was based on accurate mass measurement with an error tolerance of less than 5 ppm and LC retention time compared to standards (92% confidence level). Quantification was performed using the extracted ion chromatogram for each compound with 50 mDa windows. The linear dynamic range was determined by injecting mixtures of internal and natural standards, as indicated above. Sphingolipids: (ceramide, Cer; dihydroceramide, DHCer; sphingomyelin, SM; dihydrosphingomyelin, DHSM; hexosylceramide, HexCer; ceramide Dihexoside, CDH). Glycerophospholipids: (phosphatydilcholine, PC; lyso-phosphatydilcholine, LPC; phosphatydilethanolamine, PE; lyso-phosphatydilethanolamine, LPE). Sterol lipids: (cholesterol, CHOL; cholesteryl ester, CE).

### Bioinformatic analyses

Lipid enrichment analyses were performed using LION online software (Molenaar et al. 2019). All lipids were functionally annotated by their relative log_2_ fold change value compared to the standard Batch pGag::eGFP condition. Then, results were manually annotated and classified regarding lipid origin, lipid class, and functional properties.

## Results

### Enhancement of specific productivity within the same mode of operation reduces monomer per VLP but not membrane protein per particle

**Fig. 1:**
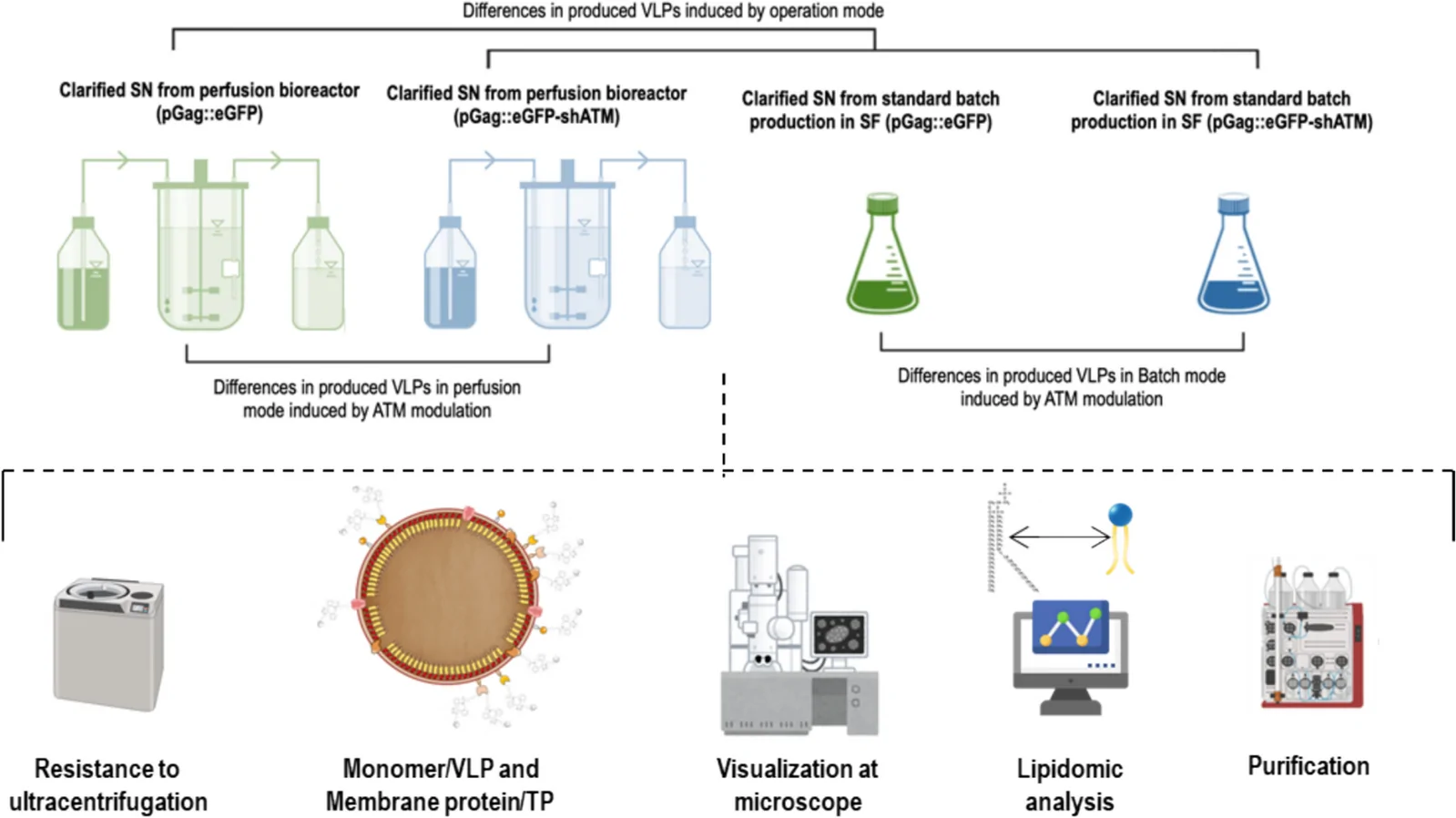
Experimental design and workflow of the experiments performed in this work. VLPs were harvested for both batch and perfusion productions and then subjected to physicochemical characterization tests. Resistance to ultracentrifugation, monomer/VLP, and membrane protein/TP were quantified. Structural differences were assessed using Cryo-TEM. Furthermore, lipidomic analysis of ultracentrifuged VLP samples was done for each studied group. A final purification comparison study was performed for supernatants coming from perfusion. VLP, virus-like particle; TEM, transmission electron microscopy

**Fig. 2:**
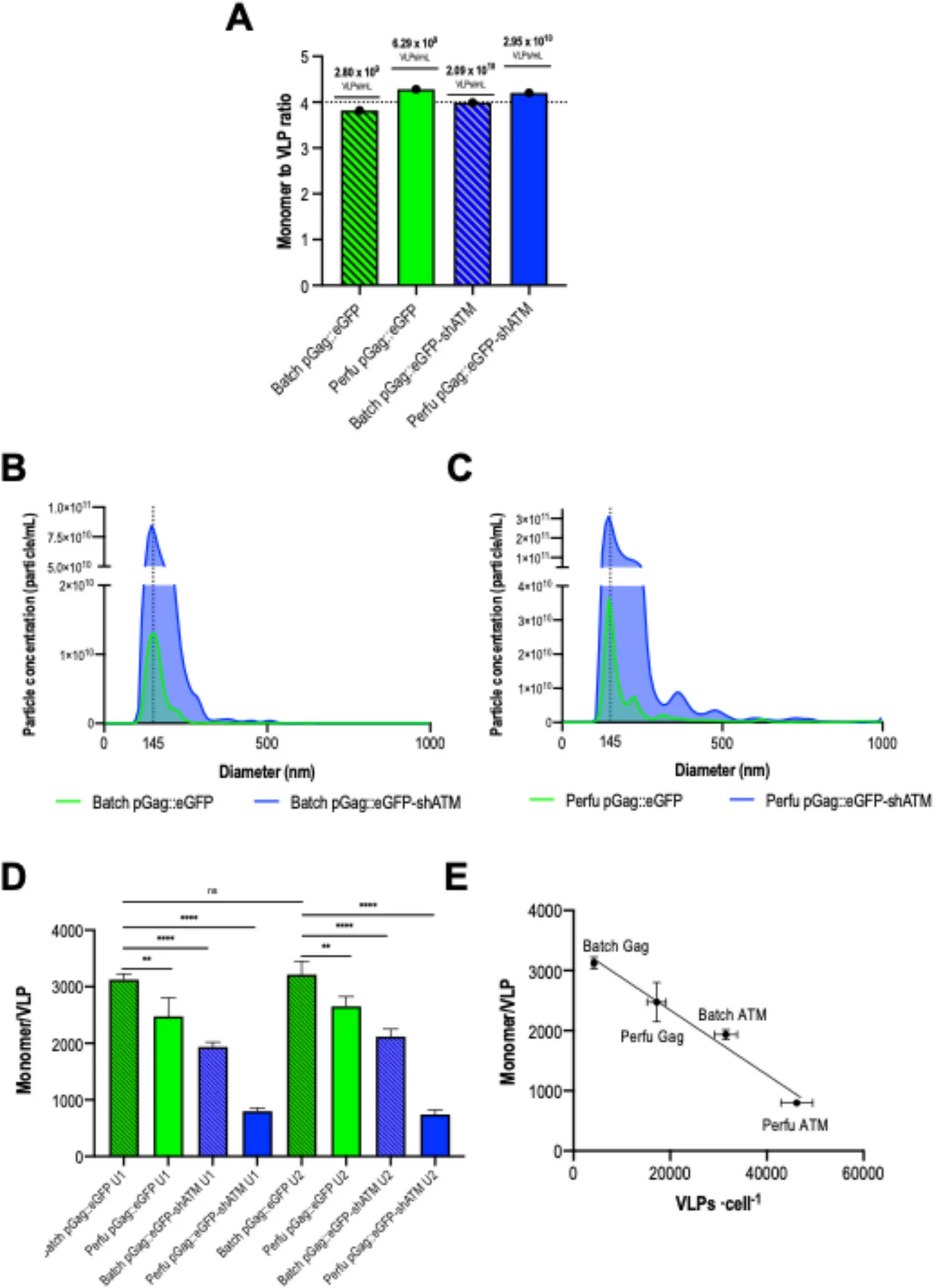
Summarized data upon monomer/VLP determination for each group. **A** Free unassembled Gag monomer to VLP ratio for each studied condition. VLP concentrations are directly measured by NTA. **B** Size and particle distribution of VLPs produced in batch measured by NTA. **C** Size and particle distribution of VLPs produced in perfusion measured by NTA. **D** Monomer/VLP ratio of each studied group calculated by ELISA. **E** Correlation between monomer/VLP and average specific production calculated for each study group. Significance was calculated using one-way ANOVA and Tukey’s test. NTA, nanoparticle tracking analysis; VLP, virus-like particle; ELISA, enzyme-linked immunosorbent assay

A complete VLP characterization study was conducted as described in Fig. 1 to gain insights upon their biochemical characteristics when produced in batch and perfusion. Moreover, this work also explored the effects of increased specific productivity by means of gene silencing strategies resulting in a quadruple comparison. We employed shRNA-mediated knockdown of the ATM (shATM) gene to enhance productivity. A fourfold increase in VLP production was observed by NTA for conditions transfected with shATM. Nonetheless, the ratio of free unassembled to assembled monomer remained approximately 4:1 for all conditions (Fig. 2A), as previously reported (Lavado-García et al. 2021b). In this context, free unassembled monomers refer to the excess Gag::eGFP molecules not incorporated into VLPs, inferred from bulk quantification data rather than directly detected as soluble protein in the medium. These free unassembled monomers can be released to the extracellular medium upon cell death. The ratio calculation arises from the theoretical VLP quantity obtained by spectrofluorimetric measurements and VLP titers quantified by NTA. Briefly, after single sucrose cushion ultracentrifugation to remove all free monomers, values for total p24 concentration were obtained by ELISA. VLP concentration was measured by NTA. Monomer/VLP ratio was then calculated using previously reported correlations (Lavado-García et al. 2021b). An estimated 3100 ± 100 monomers/VLP were obtained for the standard batch production with pGag::eGFP (Fig. 2D). This number significantly decreased (*p-*value ≤ 0.01) for the corresponding perfusion production that averaged 2500 ± 100 monomers/VLP. A severe decrease of the number of monomers could be observed (*p-*value ≤ 0.0001) for the ATM conditions where 1900 ± 100 and 800 ± 60 monomers/VLP were obtained for the batch and perfusion, respectively. The assay was performed either after one or two ultracentrifugation steps to ensure that free unassembled Gag monomer was not interfering in the monomer/VLP ratio estimation. Nonetheless, no significant differences were obtained (Fig. 2D), suggesting that free unassembled Gag monomers were being properly removed during the ultracentrifugation steps. Despite the great differences in the monomer/VLP ratio, produced VLPs from all conditions had a mean diameter of 145 nm (Fig. 2B, C), indicating that the structural differences were not critical for Gag::eGFP VLP formation. Interestingly, monomer/VLP showed a linear negative correlation with the cell-specific production of each studied group (Fig. 2E). This suggested that enhancing specific productivity could lead to reduced Gag content/VLP.

**Fig. 3:**
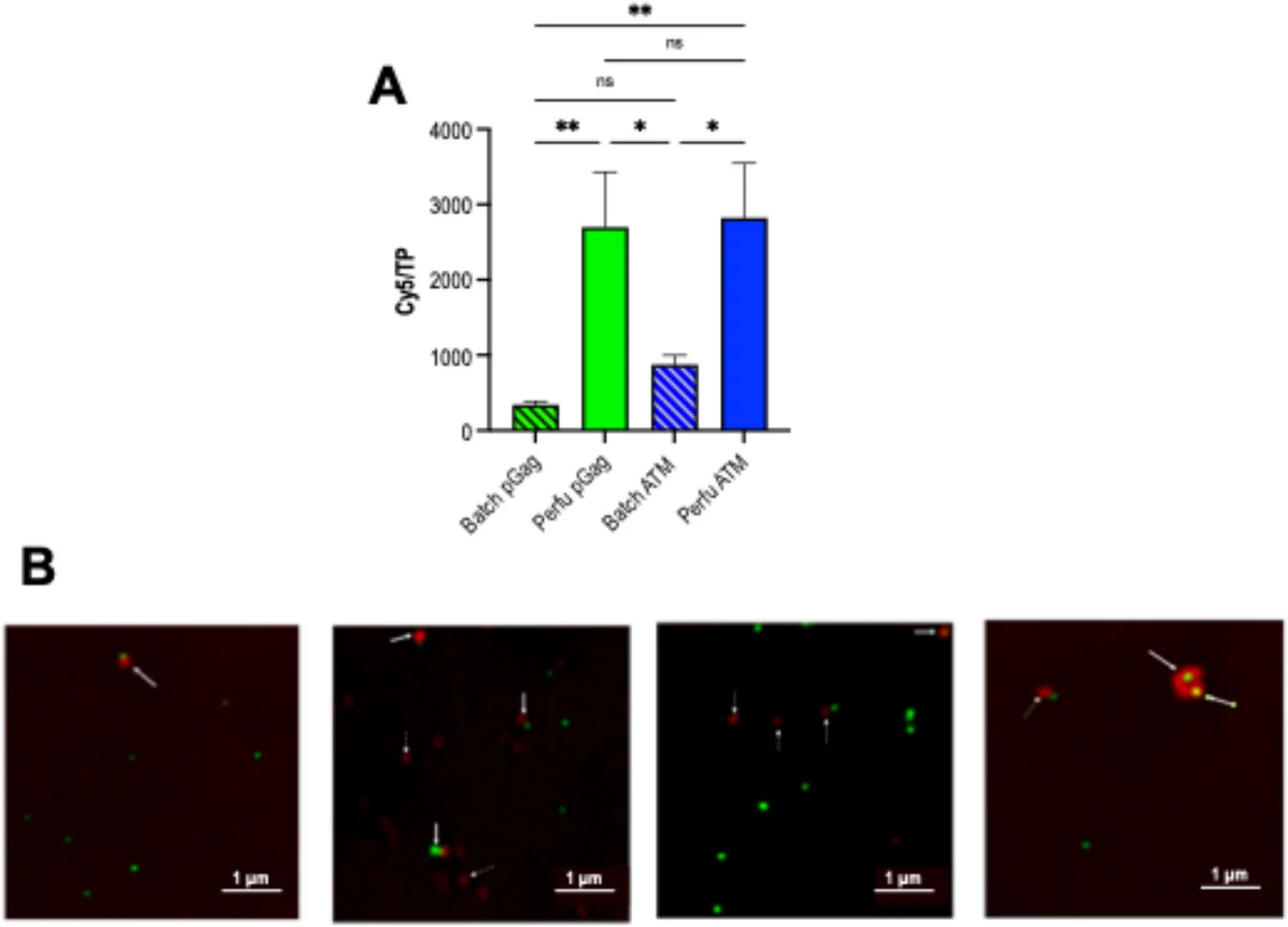
Particle functionalization.** A** Click chemistry assay for each studied group expressed as Cy5 molecules/total particle. In this case, click chemistry is used as an approximation of membrane proteins/TP. **B** Compilation of pictures taken by SRFM for each Cy5 functionalized group, where red and green fluorescence correspond to Cy5 and VLPs, respectively. VLP, virus-like particle; EV, extracellular vesicle; TP, total particles; SRFM, super resolution fluorescence microscopy

Consequently, if the number of monomers decreases as specific productivity increases, and the size remains constant, VLP monomer density decreased as a cause of the prior observations. The same concentrated VLPs by ultracentrifugation were subjected to a functionalization test using copper-free click chemistry. Since the method used relies upon membrane protein content, reagents were adjusted to both VLPs and EVs, considering a total number of particles (TPs) of 5 × 10^11^ TP/mL (Fig. 3A). Functionalization rates normalized by total particle content were assessed after a second ultracentrifugation step to remove non-bounded Cy5 (García-Trujillo et al. 2025). TPs produced by both perfusion runs achieved a functionalization rate of 2800 Cy5 molecules/TP (Fig. 3A). This value significantly dropped (*p-*value ≤ 0.01) to approximately 800 Cy5 molecules/TP and 400 Cy5 molecules/TP using nanoparticles coming from batch ATM and standard transfections, respectively (Fig. 3A). Functionalization of nanoparticles was corroborated by direct observation of the samples under the super resolution fluorescence microscopy (SRFM) (Fig. 3B). Importantly, azide-alkyne click chemistry is used for the cycloaddition of Cy5 to all free and accessible amine groups without modifying nanoparticle properties (Yi et al. 2018). Since VLPs are enveloped nanoparticles, these amine groups are coming from membrane proteins attached to the surface of the lipidic membrane. Moreover, it must be considered that all samples contain both VLPs and EVs, and the latter ones are preferentially functionalized over VLPs. Therefore, the observed functionalization is mainly due to the presence of EVs, as observed in Fig. 3B. Interestingly, the observed decrease in monomer/VLP ratio was not detected for membrane proteins/TP.

### Lipidomic profiling of VLPs and coproduced vesicles reveals decreased quantity of lipids per nanoparticle as specific productivity increases

**Fig. 4:**
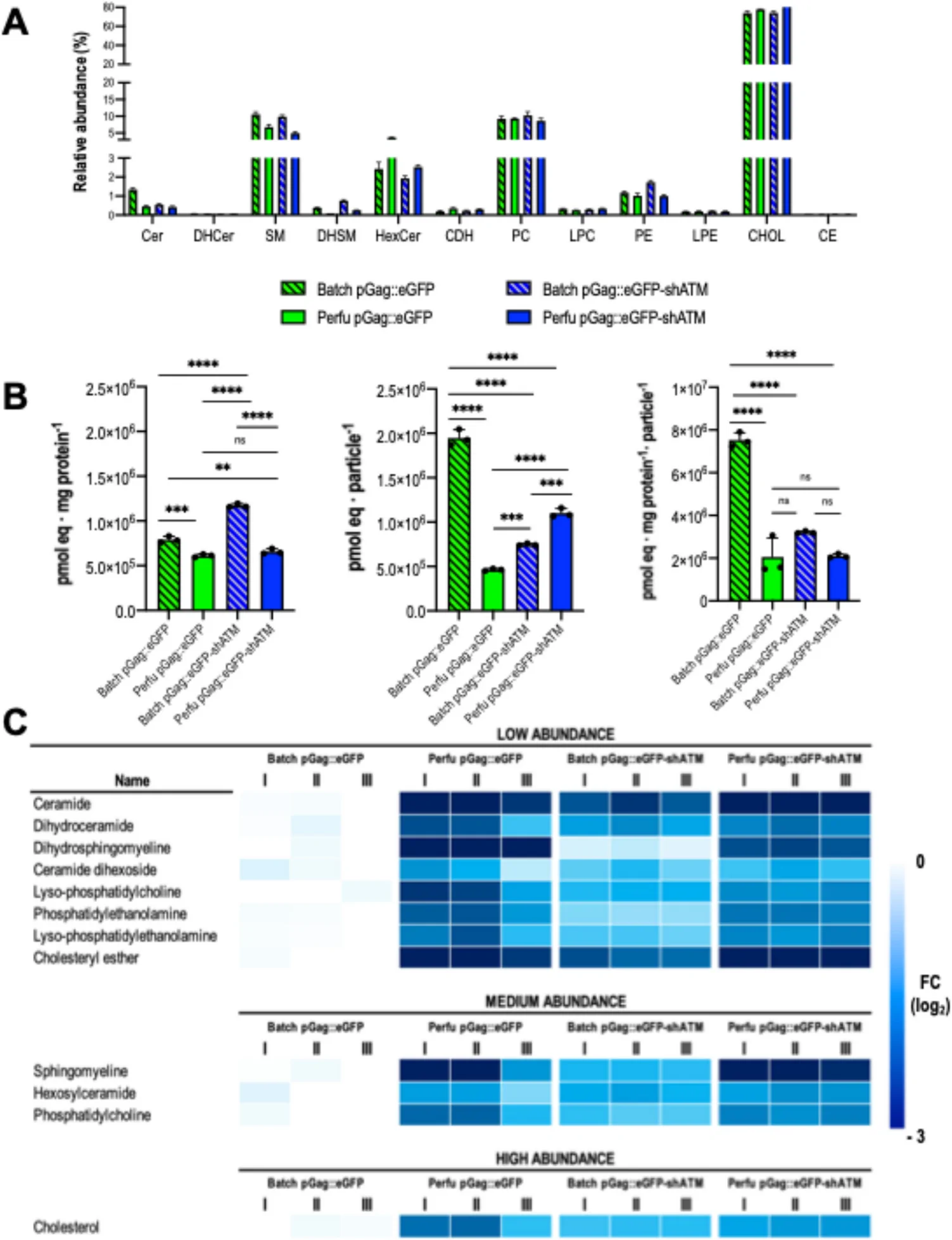
Lipidomic study performed upon ultracentrifugation for all four groups. **A** Relative abundance of all studied lipids expressed as a percentage of each quantified lipid in respect to the total sum of lipids for each sample.** B** Total quantified lipids measured in pmol equivalent normalized by protein and/or number of particles.** C** Fold change (FC) of each studied lipid in respect to batch Gag condition. Fold changes were normalized as log_2_ considering batch Gag as control. Lipids were arbitrarily classified depending on their relative abundance in three main groups depicted above. Significance was calculated using one-way ANOVA and Tukey’s test

An absolute lipidome quantification was conducted for each condition with purified and concentrated VLP samples coming from ultracentrifugation to further characterize each studied group. Cholesterol was the most prevalent lipid, observed with 80% of relative abundance (Fig. 4A). It was followed by sphingomyelin, hexosylceramide, and phosphatidylcholine with abundances ranging from approximately 5 to 15% (Fig. 4A). The rest of the detected lipids were found in abundances lower than 3% (Fig. 4A). All lipidomic profiling results were then classified depending on their relative presence in three main groups: low, medium, and high abundance (Fig. 4C). Interestingly, batch shATM showed higher amounts of total lipids (*p-*value ≤ 0.0001) than the rest of the studied groups when normalizing by the amount of loaded protein (Fig. 4B).

Completely opposite results were obtained when total lipid quantification was only normalized by the number of total particles (Fig. 4B) where the standard pGag::eGFP batch showed the highest value (*p-*value ≤ 0.0001). These results again suggested a negative correlation between the protein quantity and the number of total particles, which is especially noticeable for the batch shATM condition, corroborating the prior observation for monomer/VLP. Perfusion conditions did not seem to be highly altered by any of the normalizations. Therefore, to perform the appropriate comparative analyses, results were normalized per protein amount and number of particles (Fig. 4B, Supplementary Fig. S1). To investigate the impact of each modification upon the specific lipidomic profiling, log_2_ fold changes (FC) were calculated concerning the standard pGag::eGFP batch condition. Overall, all detected lipids were downregulated for all three conditions (log_2_ FC < 0) (Fig. 4C). On the one hand, both conditions operated in perfusion had a pronounced decrease (log_2_ FC =  − 3) in ceramide, dihydrosphingomyelin, cholesteryl ester, and sphingomyelin content, which was less noticeable for the batch shATM condition. On the other hand, lipidomic profiling of batch shATM showed a decline in its lipid content that may be attributed to enhanced particle productivity. The decrease is particularly stressed in the ceramide and cholesteryl ester content (log_2_ FC = −2) (Fig. 4C). Pairwise enrichment analyses for each dataset show significant changes in overall glycerophospholipid (GP) content (*q-*value ≥ 1*)* for all conditions (Supplementary Fig. S2). Interestingly, mitochondrial lipidic content was significantly enriched for the batch pGag::eGFP-shATM condition (*q-*value ≥ 1*)* (Supplementary Fig. S2A) whereas for both conditions involving perfusion, endoplasmic reticulum-related lipids were significantly enriched (*q-*value ≥ 1*)* (Supplementary Fig. S2 B-C). The specific decrease in lipid content of all nanoparticles was overall associated with the increase in the enrichment of lipid-related biological processes terms and their corresponding *q-*values related to particle stability and charge (Supplementary Fig. S2). This effect is particularly highlighted in the batch shATM condition, where almost all physicochemical properties *q-*value*s* are over 2, suggesting a higher degree of significance.

In line with the previous results, an overall decrease in the lipidic content was detected. Surprisingly, this happened either by a medium replacement (MR)-induced effect in perfusion or by the enhancement of specific productivity in shATM conditions.

### Physical properties of VLPs are not altered despite their biochemical differences

All four studied groups greatly differed in terms of monomer/VLP, Cy5/TP ratio, and lipid·protein-^1^·particle^−1^ ratio. To investigate if these features affected VLP integrity and quality upon purification, VLPs were subjected to diverse tests. Following two rounds of ultracentrifugation, Cryo-TEM images of each studied group were taken to assess ultrastructural differences by visualization (Supplementary Fig. S3). Despite the reduced monomer/VLP ratio, no clear differences could be observed. Differences in VLP and EV loss appeared when samples were ultracentrifuged (Table 1). Interestingly, when normalized by particle concentration, similar percentages of VLP loss were obtained (Table 2). After one ultracentrifugation, 53% and 58% of VLP loss could be measured for the batch pGag::eGFP and batch shATM conditions, respectively (Table 2). This value was reduced to ~ 45% for both perfusion conditions. Co-produced extracellular vesicles of both pGag::eGFP-shATM processes seemed to be more resistant to ultracentrifugation, considering that no loss was observed for the batch condition and only 22% for the perfusion one. Total VLP and EV loss after the second ultracentrifugation was similar for all studied conditions (Table 2), surpassing 95% of total loss in all cases. Therefore, this suggested that high particle concentrations protected VLPs from degradation when ultracentrifuged while their integrity remained the same within the same mode of operation, regardless of the enhanced specific productivity. Nevertheless, the high particle loss observed may also reflect suboptimal sedimentation conditions. Specifically, the use of a 30% sucrose cushion, although commonly employed for Gag-based VLP purification (García-Trujillo et al. 2025), could hinder the recovery of vesicles with lower densities. Future work should consider testing lower sucrose concentrations to improve recovery efficiency without compromising particle integrity. 

**Table 1:** VLP and EV loss upon ultracentrifugation

	Perfu pGag::eGFP			Perfu pGag::eGFP-shATM			Batch pGag::eGFP			Batch pGag::eGFP-shATM		
	1st ultra	2nd ultra	Total	1st ultra	2nd ultra	Total	1st ultra	2nd ultra	Total	1st ultra	2nd ultra	Total
**VLP loss (%)**	49.21	60.42	**86.66**	0	76.29	**76.29**	55.96	83.14	**95.10**	54.11	70.58	**86.50**
**EV loss (%)**	66.96	53.24	**84.55**	24.38	55.86	**66.62**	95.26	71.62	**99.10**	83.26	39.58	**93.25**

**Table 2:** VLP and EV loss upon ultracentrifugation after particle normalization

	Perfu pGag::eGFP			Perfu pGag::eGFP-shATM			Batch pGag::eGFP			Batch pGag::eGFP-shATM		
	1st ultra	2nd ultra	Total	1st ultra	2nd ultra	Total	1st ultra	2nd ultra	Total	1st ultra	2nd ultra	Total
**VLP loss (%)**	46.62	94.07	**96.83**	43.68	95.10	**97.24**	53.95	89.95	**98.10**	58.59	95.42	**95.37**
**EV loss (%)**	76.48	95.74	**99**	21.78	94.78	**95.66**	89.38	95.81	**93.94**	0.00	95.78	**99.56**

**Fig. 5:**
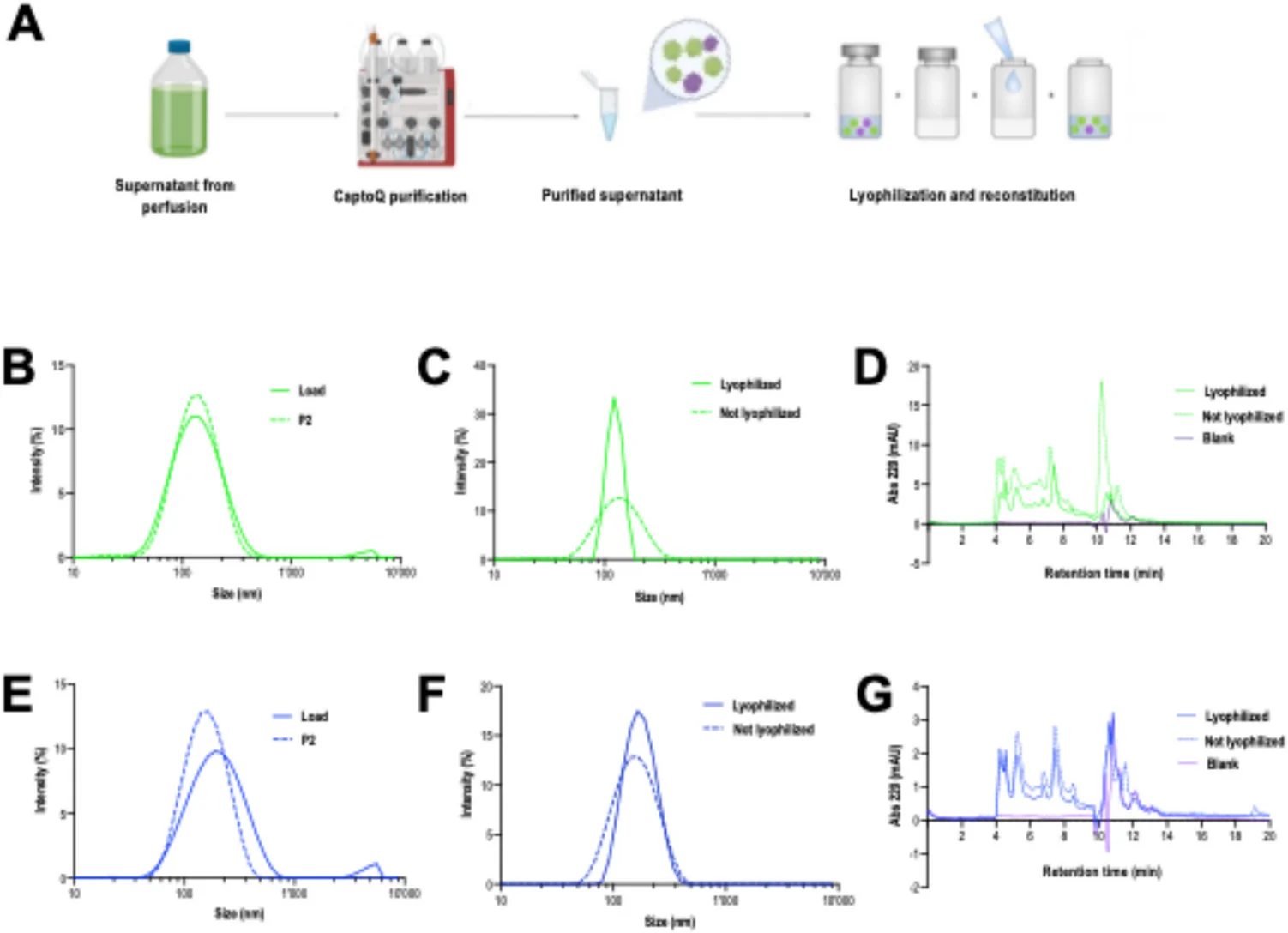
VLP purification. **A** Schematic representation of the workflow conducted from purification to lyophilization. **B** DLS profile of the pGag::eGFP-loaded supernatant and eluted fraction containing a major nanoparticle population at 140 nm. **C** DLS profile of the purified pGag::eGFP eluted fraction and lyophilized sample containing a major nanoparticle population at 140 nm. **D** HPLC profile of the purified pGag::eGFP VLP fraction and its corresponding lyophilization. **E** DLS profile of the pGag::eGFP-shATM-loaded supernatant and eluted fraction containing a major nanoparticle population at 140 nm. **F** DLS profile of the pGag::eGFP-shATM-loaded supernatant and eluted fraction containing a major nanoparticle population at 140 nm. **G** HPLC profile of the purified pGag::eGFP-shATM VLP fraction and its corresponding lyophilization. VLP, virus-like particle; DLS, dynamic light scattering; HPLC, high performance liquid chromatography; Abs, absorbance

Finally, two purification runs were conducted with the clarified supernatants coming from both perfusions to evaluate any impact that the differential biochemical properties may have upon the interactions with chromatographic columns (Fig. 5, Supplementary Figs. S4, S5). Quality attributes after the chromatographic purification were exclusively tested for VLPs obtained from perfusion runs. This choice was made because the cell retention device for the perfusion run served as the initial clarification step for the complete integrated process of continuous VLP production. For this purpose, an anionic exchanger column (AEX), CaptoQ, was used to conduct a primary purification followed by lyophilization (Fig. 5A**)**. For both cases, the purification yield was calculated to be at 50% (Tables 3 and 4). Mean particle diameter after CaptoQ purification was at 140 nm for the two perfusions (Fig. 5B–E) and was maintained after lyophilization (Fig. 5C–F). VLP stability was observed under Cryo-TEM before and after lyophilization for both processes (Supplementary Figs. S4E—S5E) coherently showing well-assembled VLPs. Interestingly, the standard pGag::eGFP perfusion reduced the degree of polydispersity in nanoparticles after lyophilization. This unveils the great potential of including a lyophilization step for VLP-based vaccines as their stability does not seem to be compromised (González-Domínguez et al. 2021). This was then corroborated by comparing the HPLC profiles of each purified sample to the corresponding lyophilization, displaying little to no variation for both cases (Fig. 5D–G).

**Table 3:** Data collection for the purification of the perfusion supernatant of pGag::eGFP

	**Total protein and dsDNA contaminant removal**					**Gag::eGFP-VLPs recovery and purity**			
Sample	Volume (mL)	Total protein (µg/mL)	Step removal (%)	dsDNA (ng/mL)	Step removal (%)	Total Gag::eGFP-VLPs (by NTA)	Step recovery (%)	Purity (%) (VLPs/TPs)	Enrichment (%) (VLPs/TPs)
Load	25	1,272.7	-	1,519.0	-	1.35 × 10^11^	100	31	-
Elution	5	260.2	96	1,896.1	90	7.33 × 10^10^	54	31	0

**Table 4:** Data collection for the purification of the perfusion supernatant of pGag::eGFP-shATM

	Total protein and dsDNA contaminant removal					Gag::eGFP-VLPs recovery and purity			
Sample	Volume (mL)	Total protein (µg/mL)	Step removal (%)	dsDNA (ng/mL)	Step removal (%)	Total Gag::eGFP-VLPs (by NTA)	Step recovery (%)	Purity (%) (VLPs/TPs)	Enrichment (%) (VLPs/TPs)
Load	7	1,911	-	6,828.2	-	2.12 × 10^11^	100	31	-
Elution	6	76.4	97	257.3	97	1.07 × 10^11^	50	47	16

To sum up, differences in ultracentrifugation sensitivity could be determined between batch and perfusion-derived VLPs but not induced by ATM silencing. No differences could be observed in the degradation and purification yield for the studied conditions, suggesting that the induced biochemical modifications had little impact upon VLP integrity.

## Discussion

Despite the high biological complexity of VLPs, characterization techniques usually rely solely upon particle diameter determination and co-contaminant estimations (Cervera et al. 2019). These techniques have high screening capacity, yet important biochemical attributes are not often characterized. Quality control of the produced VLPs requires deeper knowledge of the number of Gag molecules, lipids, and membrane proteins per particle. This work comprises a complete VLP and co-produced particle characterization by easy-to-implement techniques for specific monomer, membrane protein, and lipid determination, allowing for accurate and fast assessment of potential CQAs.

**Fig. 6:**
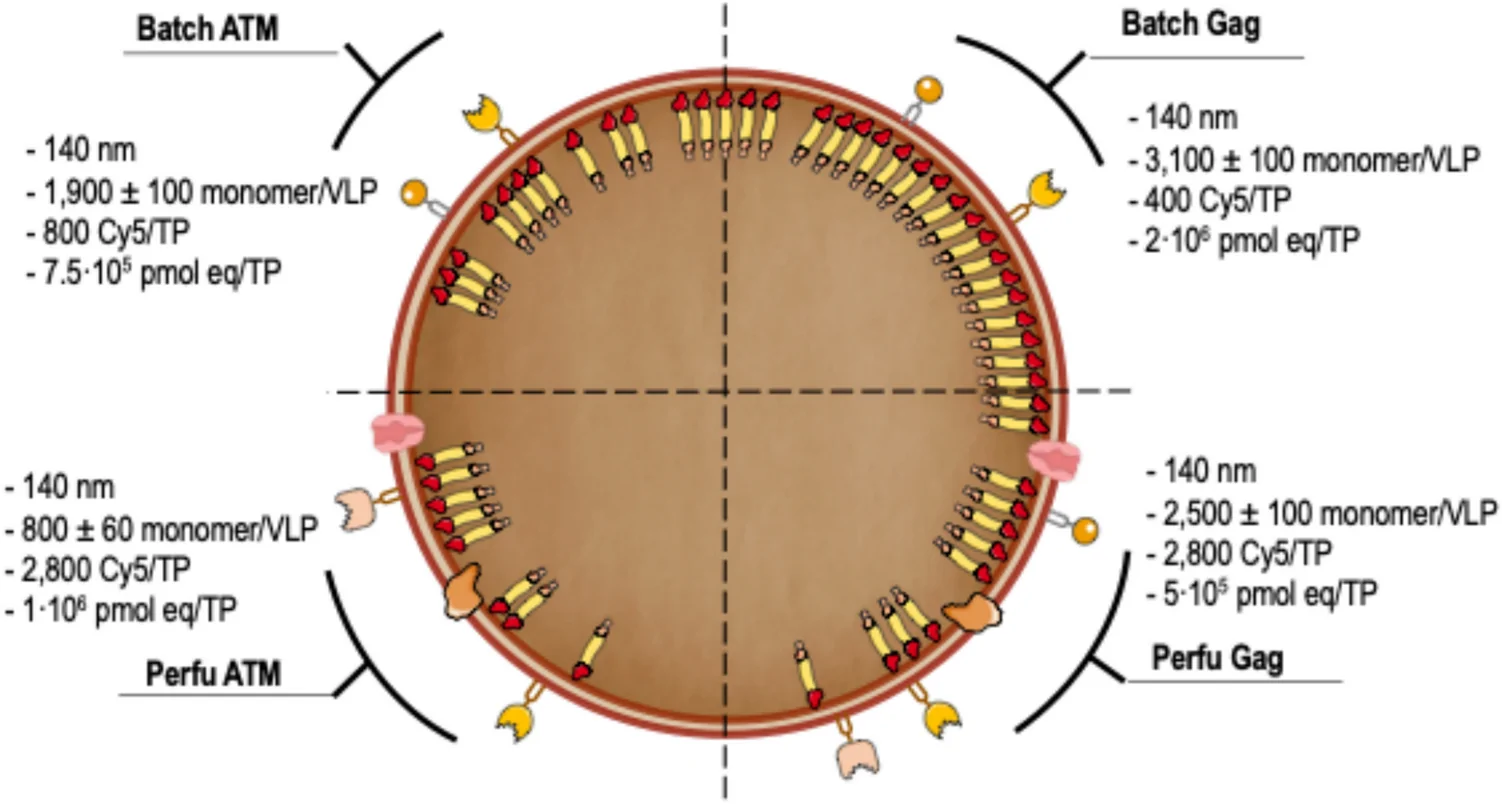
Schematic representation of the physicochemical parameters for all studied VLPs. This is a simplification of the internal structure of the VLPs. It does not imply the presence of a regular Gag lattice. It serves as a conceptual aid rather than a structural model.TP, total particle; VLP, virus-like particle; eq, equivalent (pmol of equivalent normalized by number of total particles)

Recent studies focused on Gag stoichiometry determination hypothesized that increased particle production between cell lines may be the underlying reason for a decrease in the specific monomer ratio (Lavado-García et al. 2021b). Here, we explored this hypothesis by increasing specific particle production using metabolic or bioprocess engineering strategies in HEK293 cell cultures. A rapid decline in the specific monomer content was observed when specific productivity was enhanced either by metabolic or bioprocess engineering approaches, corroborating the initial hypothesis. Likewise, we hypothesized that the increase in particle production would also be reflected in the quantity of membrane proteins per TP (Lavado-García et al. 2022a, b). To test so, we utilized a Cy5-based click chemistry approach (García-Trujillo et al. 2025). Interestingly, those particles (both VLPs and EVs) produced in perfusion yielded significantly higher values of functionalization. Moreover, the induced increase in specific productivity by the silencing of ATM did not alter the rate of functionalization. Increased productivity could enhance the rate of budding and potentially lead to a dilution of membrane proteins if the cell does not replenish them quickly enough. In this scenario, even with similar particle size, an increased budding rate might affect functionalization. However, the same ratio for protein/TP suggests a different explanation. Importantly, this click chemistry approach has been reported to preferentially react with EVs, being these nanoparticles 100% functionalized. In fact, only 4% of VLPs have been shown to be functionalized with this methodology (García-Trujillo et al. 2025). Therefore, we cannot conclude if VLPs change their membrane protein density, as the EV population completely masked the functionalization measurements. However, based on the TP production, we hypothesize that the inclusion of continuous MRs in the perfusion mode allows the enhancement of membrane protein density for the manufacturing of EVs. Consistently, previous glycomic studies quantifying *N*- and *O*-glycosylations upon Gag VLP generation showed that EVs contained more glycans—and therefore membrane protein density—than VLPs (Lavado-García et al. 2022a, b). A final lipidomic study provided insights upon particle formation for each condition. Coherently, specific lipidic content was also reduced either induced by a MR effect or by ATM silencing. Moreover, cholesterol has been recently identified as one of the main lipids that form nanodomains for Gag assembly and excision (Favard et al. 2019). In this study, cholesterol has been shown to be the most abundant lipid, making it a potential target for media supplementation to improve Gag-based VLP assembly and production (Lavado-García et al. 2020b). Notably, the observed decrease in lipid content per particle, while size remained constant, suggests a reduction in membrane lipid density rather than lipid internalization, as the extraction method primarily targets membrane-associated lipids. Although this effect was observed across several lipid classes, further studies comparing these profiles with VLPs produced using only Gag (Gag VLP lacking GFP) would be valuable to clarify the specific influence of the GFP tag on lipid incorporation. Therefore, the mentioned results suggest that Gag transportation to the cell membrane and its assembly are biological processes that occur at lower speeds compared to vesicle excision. These means that metabolic engineering strategies to enhance Gag VLP quality should firstly target the increase in monomer transportation and assembly-related processes (Lavado-García et al. 2021a). The recent improvements in the generation of stable cell lines for Gag-based VLP production could lead to the implementation of inducible systems (Szenk et al. 2020) to control and tune the specific productivity to achieve the optimal production rate to have the desired CQAs. The generation of HEK293 stable cell lines overexpressing ESCRT-related proteins would also help tackle this problem (Meng et al. 2015). Unlike Gag transportation, the rate of membrane protein renewal was similar to the EV production rate, even when cell-specific productivity was increased. In fact, perfusion-based strategies were found to be better in this aspect, suggesting that MR allows for increased membrane protein turnover, thus enabling higher rates of functionalization. This holds special interest if these nanoparticles were to be used as drug delivery carriers or as click chemistry-based vaccine platforms. Their enhanced functionalization rate would portend higher potential as nanocarriers or vaccine platforms if the VLPs were decorated with an epitope of interest (Nooraei et al. 2021; He et al. 2022). Stably enhancing the rate of membrane protein renewal in HEK293 cell cultures is presented as a much more difficult task given the number of cellular interactions that must be considered. Finally, this increased secretion rate also influenced the specific lipidic content. It is worth noting that perfusion involves the use of continuous MR to extend production time and remove toxic by-products that may accumulate in the cell culture media. Considering that MRs provide fresh nutrients, it would be logical to assume that the harvested particles contain similar, if not higher, lipid content to that observed in the control condition. Still, the results obtained from the lipidomic analyses showed otherwise, indicating that a constant replenishment of the nutrient pool is not enough to counterbalance the enhanced secretion rate. Interestingly, gene ontology analysis of the main lipidic content of each sample revealed the appearance of mitochondrion-derived lipids for the batch conditions and endoplasmic reticulum-related for the perfusions (Supplementary Fig. S2). MR have been reported to stimulate the production of EVs, and these entities can be internally derived from the endoplasmic reticulum (Pérez-Rubio et al. 2024). Hence, the possible detection of endoplasmic reticulum lipids may be related to this effect caused by the continuous MR. The detection of mitochondrion lipids may be explained by an increased oxidative stress caused by ATM silencing and the derived enhancement in specific productivity (Ademowo et al. 2017; Lavado-García et al. 2020b). These differences may contribute to the enhanced ultracentrifugation sensitivity for the VLPs produced in perfusion mode. Furthermore, the reduction in lipid content was predicted to impact transition temperatures and electrostatic charges of the nanoparticles (Supplementary Fig. S2). Coherently, the purification rates of both perfusions were approximately at 50% while the reported purification rate for a VLP production coming from a batch culture in a CaptoQ column is at 70% (Lorenzo et al. 2023). Contrary to what it was expected, the degradation rate did not seem to be affected by the predicted changes in the lipidome. It is important to mention that lipid ontology analyses do not consider the protein content, nor do they take into consideration the free and associated cholesterol. Hence, some biochemical processes may be altered if these were considered. All the results presented here are summarized in Fig. 6 and point at ultrastructural differences in the 3D structure of VLPs upon variations in specific productivity and operation mode. To further characterize these differences, electron tomography or single particle analysis may serve as a tool for VLP visualization (Ewers et al. 2005; Zhao et al. 2014; Lavado-García et al. 2021b; Winter and Chlanda 2021).

Overall, this work has shown the great influence that bioprocess and genetic engineering strategies have in the biophysical properties of Gag-based VLPs. Surprisingly, VLPs produced by a standard TGE exhibited the best overall CQAs of all the studied groups, unveiling that, sometimes, the simpler the better. Therefore, further research should focus on improving the standard batch transfection by enhancing other process parameters such as the cell density, which are currently restricted by the cell density effect (CDE) (Lavado-García et al. 2022a, b).

## Conclusion

In this study, HIV-1 Gag::eGFP VLPs have been thoroughly characterized in terms of protein and lipidic content upon modulation of specific productivity and operation mode. A linear negative correlation was observed between the Gag monomers-to-VLP ratio and cell-specific productivity. The standard batch production yielded 3100 ± 100 monomers per VLP, while this value decreased to 1900 ± 100 and 800 ± 60 for the perfusion and batch ATM conditions, respectively. Additionally, the functionalization rates, measured as the Cy5 molecules per TP, decreased from 2800 Cy5 molecules for both perfusions to 800 and 400 Cy5 molecules for the batch transfections of ATM and standard condition, respectively. Full lipidome characterization revealed decreased lipidic content induced either by a MR or gene silencing effect. Furthermore, they maintained a mean diameter of 140 nm regardless of the methods used for their manufacturing. Ultracentrifugation sensitivity tests revealed differences in the produced VLPs between batch and perfusion. Purification of perfusion supernatants allowed 50% of VLP recovery and successful lyophilization. All in all, this work compiles a set of tools for routine VLP and EV quality assessment and raises awareness for potential changes in HIV-1 Gag VLP CQAs upon metabolic or process intensification strategies.

## Supplementary Information

Below is the link to the electronic supplementary material.

ESM1 (PDF 9.02 MB)

## Data Availability

No datasets were generated or analysed during the current study.
