# Supplementary material for: Impact of specific productivity and operation mode upon the biophysical properties of HIV-1 Gag-based virus-like particles
Source: Appl Microbiol Biotechnol. 2025 Aug 8;109(1):179. doi: 10.1007/s00253-025-13560-9 (PMC12334472; doi:10.1007/s00253-025-13560-9)
Supplement: Supplementary file 1 — (PDF 9.02 MB) [file 253_2025_13560_MOESM1_ESM.pdf]

Applied Microbiology and Biotechnology

Supplementary Material

Impact of specific productivity and operation mode upon the biophysical properties of HIV-1 Gag based virus-like particles

Pol Pérez-Rubio[1*], Elianet Lorenzo Romero[1*], Josefina Casas[2,3], Andy Díaz-Maneh[1], Francesc Gòdia[1], Laura Cervera[1†], Jesús Lavado-García[4†]

[1]Grup d'Enginyeria de Bioprocessos i Biocatàlisi Aplicada, Escola d'Enginyeria, Universitat Autònoma de Barcelona, Campus de Bellaterra, Cerdanyola del Vallès, 08193 Barcelona, Spain

[2]Research Unit on Bioactive Molecules (RUBAM), Department of Biological Chemistry, Institute for Advanced Chemistry of Catalonia (IQAC-CSIC), Spanish National Research Council (CSIC), 08304 Barcelona, Spain

[3]Centro de Investigación Biomédica en Red de Enfermedades Hepáticas y Digestivas (CIBEREHD), Instituto de Salud Carlos III, 28029 Madrid, Spain

[4]Group of Mammalian Cell and Bioprocess Engineering, Novo Nordisk Foundation Center for Biosustainability, DTU Biosustain, Technical University of Denmark, 2800 Kgs. Lyngby, Denmark

*These two authors contributed equally to this work

†Corresponding Authors:

Laura Cervera (e-mail: laura.cervera@uab.cat)

Jesús Lavado-García (e-mail: jlavgar@dtu.dk)

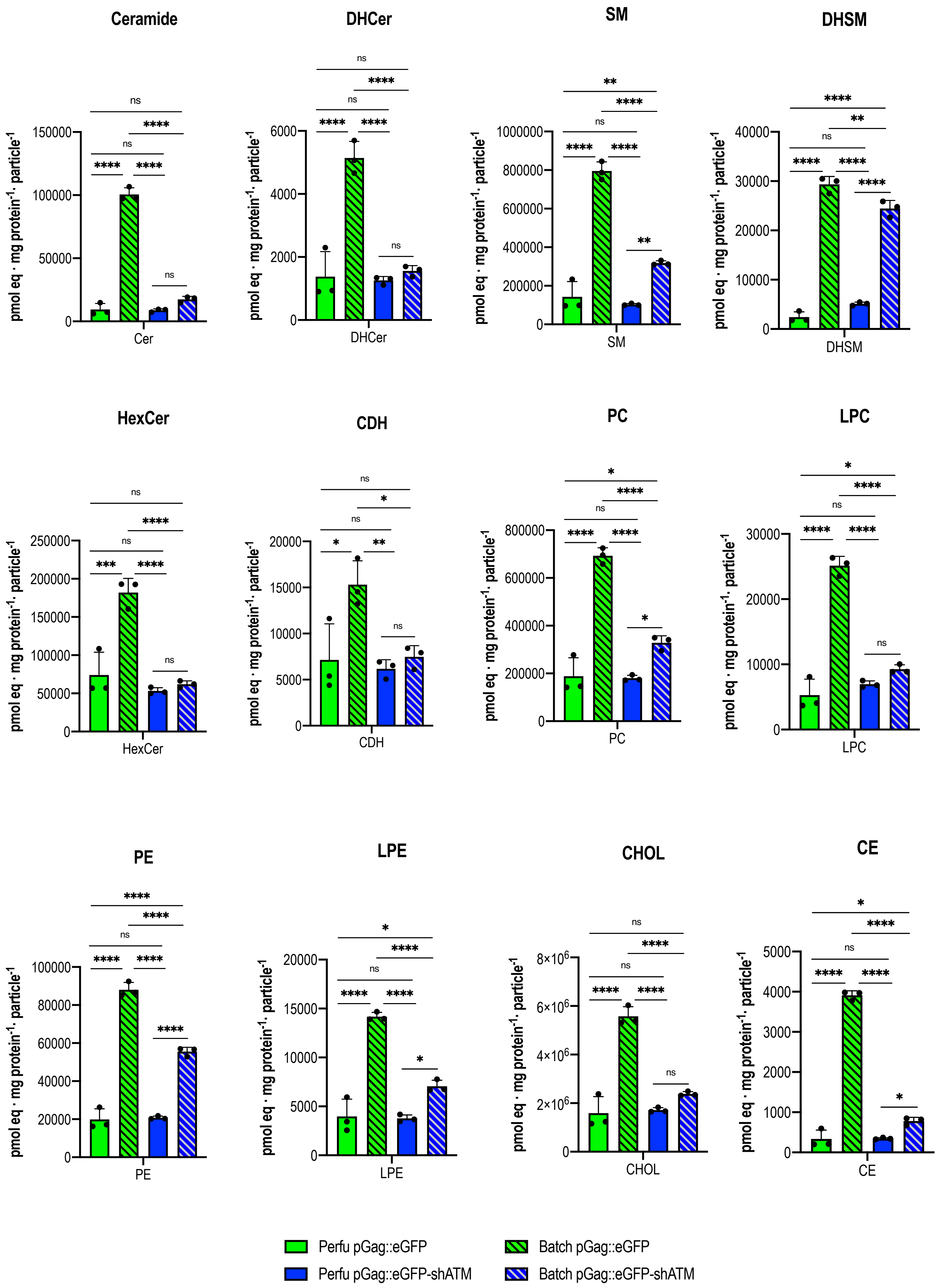

Supplementary Fig S1. Pairwise comparisons and absolute quantifications of the lipidomic study. Significance was calculated using one-way ANOVA and Tukey's test. ceramide, Cer; dihydroceramide, DHCer; sphingomyelin, SM; dihydrosphingomyelin, DHSM; hexosylceramide, HexCer; ceramide dihexoside, CDH; phosphatydilcholine, PC; lyso-phosphatydilcholine, LPC; phosphatydilethanolamine, PE; lyso-phosphatydilethanolamine, LPE; cholesterol, CHOL; cholesteryl ester, CE.

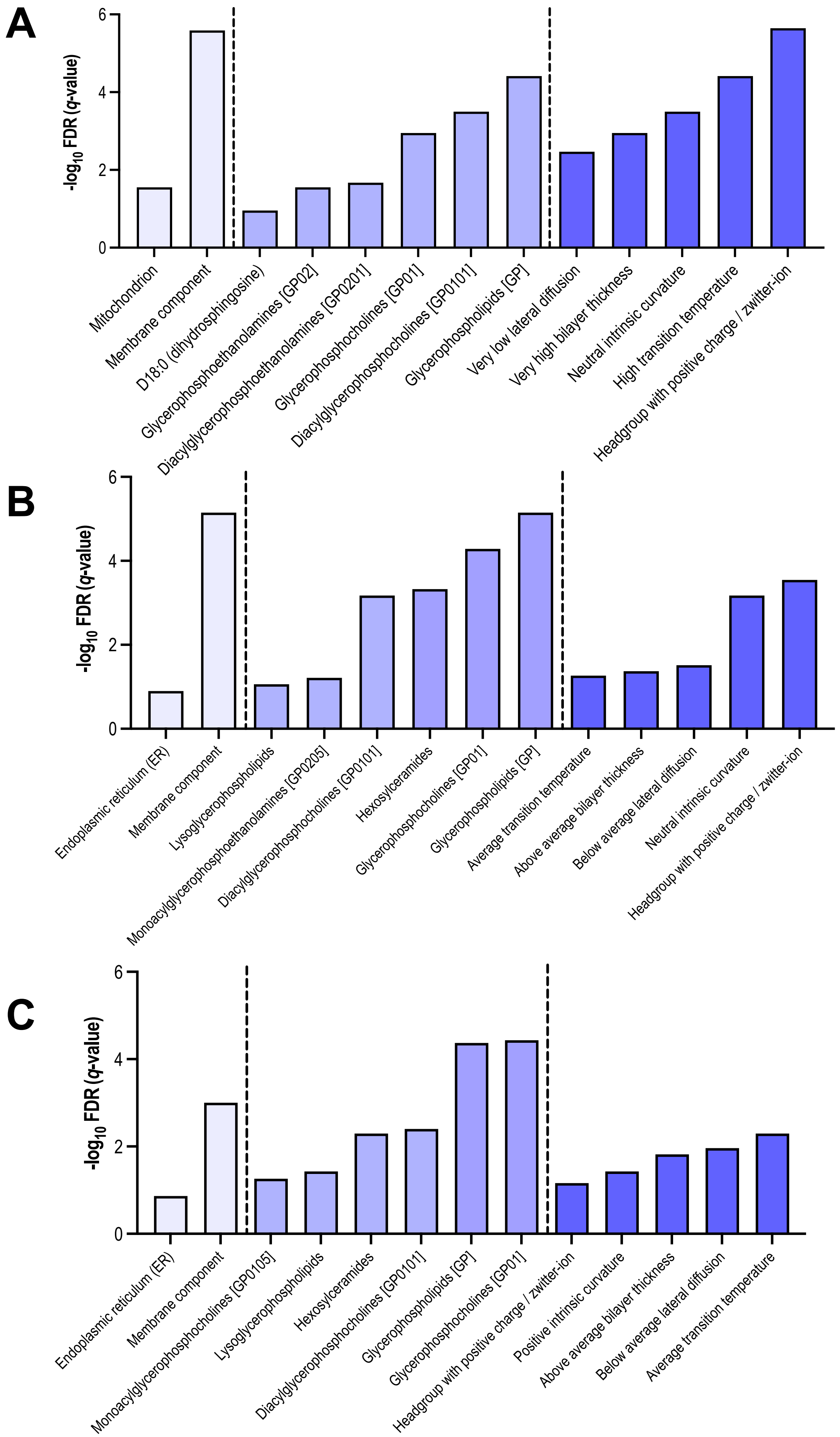

Supplementary Fig S2. Pairwise enrichment analyses performed with LION software. All lipid species were $\log_2$ normalized in respect to the Batch Gag condition. Only non-redundant significantly enriched processes are represented. Enrichment results were classified in three groups (left to right): lipid origin, lipid class and associated physicochemical properties. **A)** Pairwise comparison of batch ATM vs batch Gag. **B)** Pairwise comparison of perfu Gag vs batch Gag. **C)** Pairwise comparison of perfu ATM vs batch Gag.

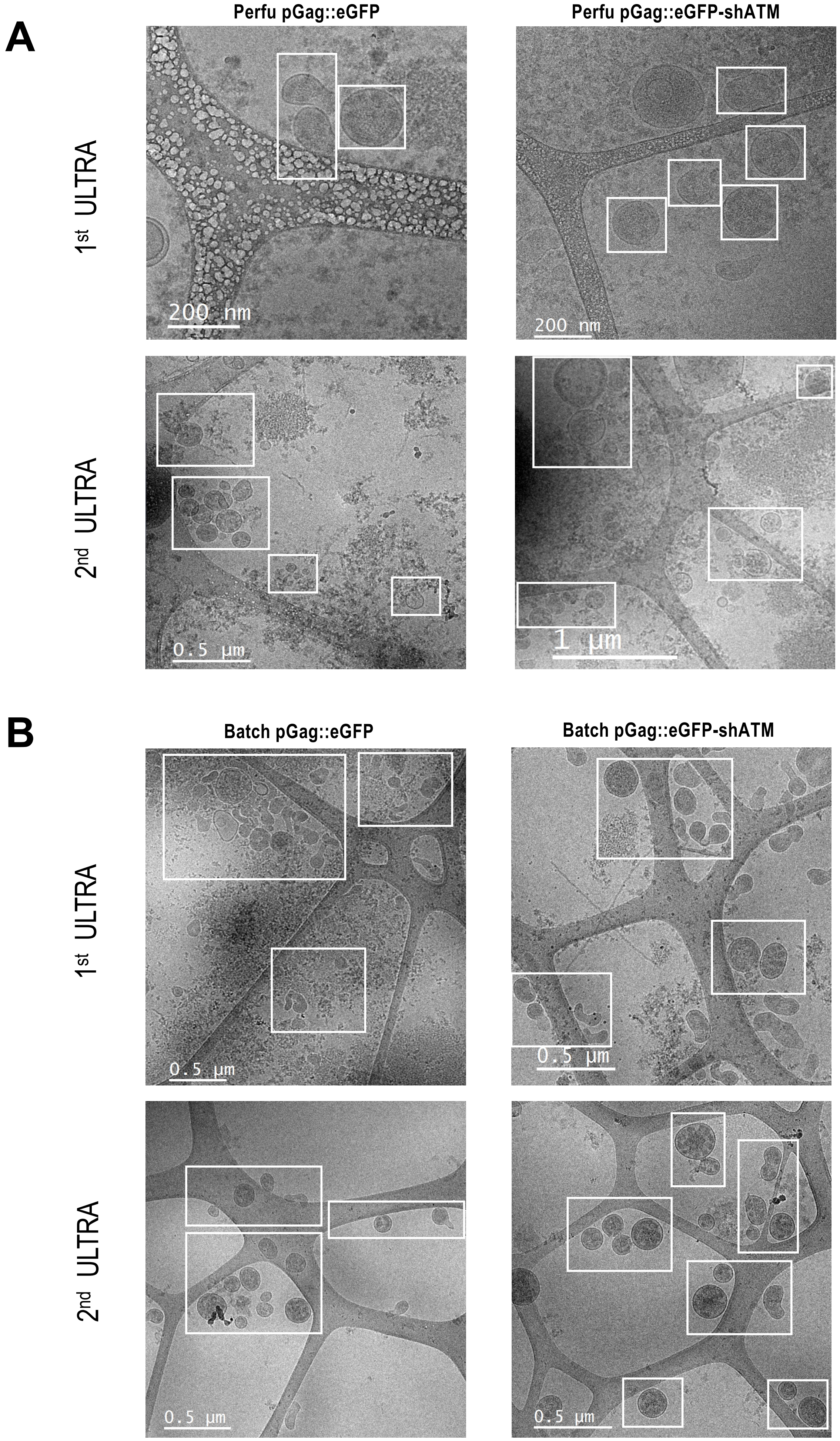

Supplementary Fig S3. Compilation of Cryo-TEM micrographs of purified VLPs by means of ultracentrifugation. **A)** VLPs produced in perfusion operation mode observed under Cryo-TEM conditions after two rounds of ultracentrifugation. **B)** VLPs produced in batch operation mode observed under Cryo-TEM conditions after two rounds of ultracentrifugation. Cryo-TEM images were obtained from samples ultracentrifuged through a 30% sucrose cushion, and background heterogeneity may reflect residual sucrose affecting vitrification quality. *VLP:* virus-like particle, *TEM:* transmission electron microscopy.

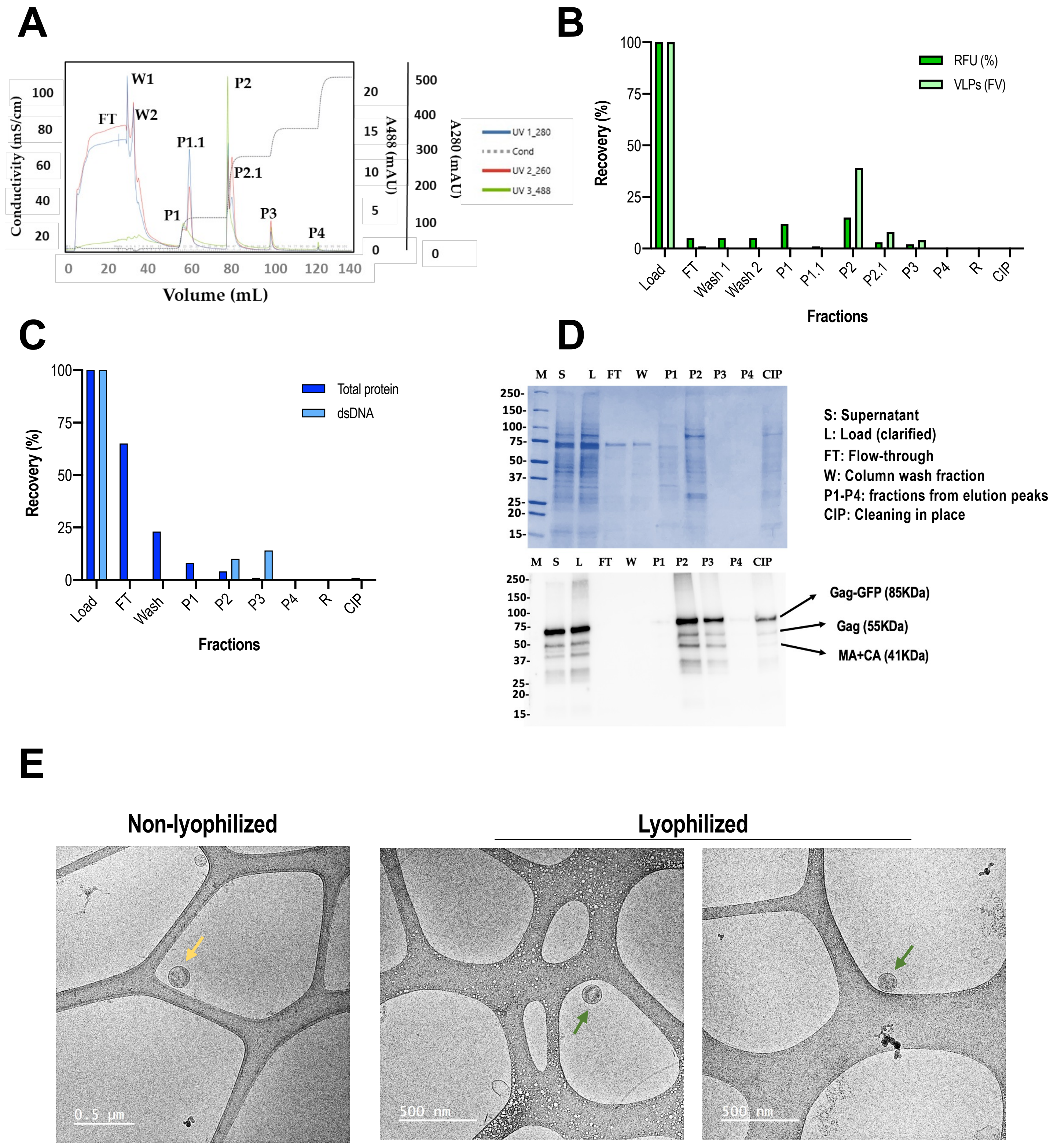

Supplementary Fig S4. Purification data corresponding to a perfusion-based process for pGag::eGFP. **A**) Chromatogram of the corresponding purification performed with an AKTA purifying system equipped with a CaptoQ column. **B)** Recovery expressed as percentage to the initial step for each corresponding purified peak. Data is calculated by means of spectrofluorometry and flow virometry. **C)** Removal of protein and dsDNA contaminants in each purified peak expressed as percentage of removal from the initial quantity. **D)** SDS-PAGE and WB analysis for each fraction showing the presence of diverse Gag::eGFP isoforms. **E)** Cryo-TEM micrographs showing the presence of well assembled Gag::eGFP VLPs both in a purified fraction and after lyophilization. *VLP:* virus-like particle, *dsDNA:* double-stranded DNA, *SDS-PAGE:* sodium dodecyl sulfate-polyacrylamide gel electrophoresis, *WB:* western blot, *TEM:* transmission electron microscopy.

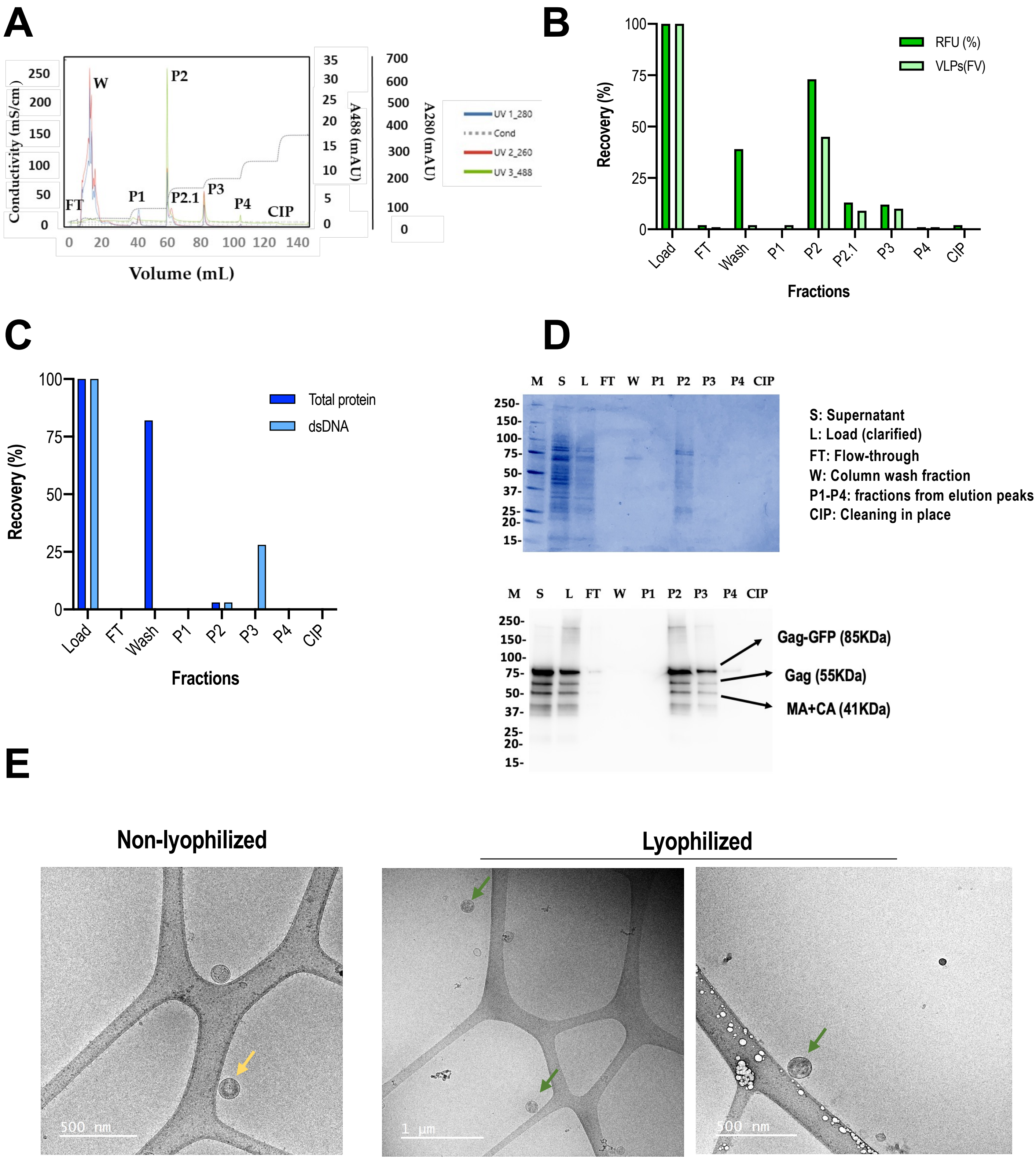

Supplementary Fig S5. Purification data corresponding to a perfusion-based process for pGag::eGFP-shATM. **A**) Chromatogram of the corresponding purification performed with an AKTA purifying system equipped with a CaptoQ column. **B)** Recovery expressed as percentage to the initial step for each corresponding purified peak. Data is calculated by means of spectrofluorometry and flow virometry. **C)** Removal of protein and dsDNA contaminants in each purified peak expressed as percentage of removal from the initial quantity. **D)** SDS-page and WB analysis for each fraction showing the presence of diverse Gag::eGFP isoforms. **E)** Cryo-TEM micrographs showing the presence of well assembled Gag::eGFP VLPs both in a purified fraction and after lyophilization. *VLP:* virus-like particle, *dsDNA:* double-stranded DNA, *SDS-PAGE:* sodium dodecyl sulfate-polyacrylamide gel electrophoresis, *WB:* western blot, *TEM:* transmission electron microscopy.
